# CT-guided ^125^I brachytherapy for recurrent ovarian cancer

**DOI:** 10.18632/oncotarget.15905

**Published:** 2017-03-04

**Authors:** Ping Liu, Lina Tong, Bin Huo, Dong Dai, Wenxin Liu, Ke Wang, Ying Wang, Zhi Guo, Hong Ni

**Affiliations:** ^1^ Department of Interventional Therapy, Tianjin Medical University Cancer Institute and Hospital, National Clinical Research Center for Cancer, Key Laboratory of Cancer Prevention and Therapy, Tianjin, People's Republic of China; ^2^ Department of Oncology, The Second Hospital of Tianjin Medical University, Tianjin, People's Republic of China; ^3^ Department of Molecule Imaging and Nuclear Medicine, Tianjin Medical University Cancer Institute and Hospital, Tianjin, People's Republic of China; ^4^ Department of Gynecologic Oncology, Tianjin Medical University Cancer Institute and Hospital, Tianjin, People's Republic of China

**Keywords:** ^125^I seeds, brachytherapy, recurrent ovarian cancer

## Abstract

This retrospective study was to evaluate the local control and survival of ^125^I brachytherapy for recurrent ovarian cancer. 52 ^125^I brachytherapy procedures were performed in 47 patients with 51 recurrent ovarian cancer lesions. The follow-up period was 1-55 months (median 12 months). The local control rate (LC) of 3, 6, 12, 24 and 36 months was 93.3%, 77.7%, 58.9%, 38.7% and 19.3%, respectively. Patients with tumor size ≤ 4cm (85.7% vs 40.0%, *P* = 0.037) and actual D90 between 110 to 130Gy (47.4% vs 66.7% vs 62.5%, *P* = 0.029) had better LC. The 1, 2 and 3 years of overall survival (OS) was 79.3%, 63.0% and 52.5%, respectively. The poor performance status (HR 3.821, 95% CI 1.383-10.555; *P* = 0.010), concurrent distant metastasis (HR 9.222, 95% CI 1.710-49.737; *P* = 0.010) and large postoperative residual tumor size (HR 6.157, 95% CI 1.438-26.367; *P* = 0.014) were closely correlated with a poor OS. Our data indicate thatCT-guided ^125^I brachytherapy is an effective and safe modality for the local treatment of recurrent ovarian cancer.

## INTRODUCTION

Ovarian cancer (OC) remains the fifth most common cause of cancer death in women [1]. Despite the intense therapeutic regimen include adjuvant platinum-based chemotherapy and optimal cytoreduction, 60%-70% patients will eventually relapse [2]. Due to this high recurrence rate, different types of adjuvant treatment have been tested. Conventional treatment for recurrent OC is chemotherapy and/or cytoreduction. However, the therapy eventually relevant to chemoresistance and it is unclear whether all patients benefit from a comprehensive surgical intervention in the same way [3, 4]. Therefore, the treatment of recurrent OC is usually challenging.

^125^I brachytherapy has been accepted as a useful and minimally invasive treatment for different tumors with significant efficacy. The most common application of ^125^I interstitial irradiation has been in the treatment of prostatic malignancies [5, 6], although therapy for other sites of disease, such as central nervous system, head-and-neck tumors, lung, hepatic and pancreatic cancer has also been described [7–12]. ^125^I brachytherapy combines the advantages of delivering a high dose of irradiation to the tumor with a very sharp fall-off outside the implanted volume; thus, sparing nearby normal tissues [13]. This is of great value especially to the recurrent OC because most patients had experienced more than one times of surgery and the anatomical structure had changed a lot. Furthermore, most of the recurrent tumors were near or adhered by momentum, vessels, ureter and urethra, vagina, cyst or rectum et al. Thus, most of the tumors could not be removed because of local extension, or inaccessibility because of the distorted geometry, or their removal would cause severe functional disability. ^125^I brachytherapy with its unique characters may address this clinical problem.

In our institution, ^125^I brachytherapy has been employed for salvage or supportive treatment of gynecological cancer in dedicated brachytherapy suits since 2010. The purpose of this study was to review and update our experience and to evaluate the significance of local treatment with ^125^I interstitial brachytherapy among patients with recurrent ovarian cancer.

## RESULTS

### ^125^I brachytherapy

A total of 52 ^125^I brachytherapy procedures were performed in 47 patients with 51 recurrent ovarian cancer lesions. Of 47 patients, 42 (89.4%) met the treatment planning system criteria after the first procedure. Five patients did not reach the treatment planning system criteria, followed by additional implantations. The final ^125^I brachytherapy achieved rate was 100%. The actuarial D90 (dose delivered to 90% of the target volume) ranged from 95-162 (mean 126Gy). The V100 (the percentage of the target volume receiving at least 100% of the prescription dose) of each patient was more than 95%, and the V150 (the percentage of the target volume receiving at least 150% of the prescription dose) for all cases was less than 50%. The D2cc for organs at risk was less than 70-75Gy. The total number of implanted seeds was 1775, with an average of 35.6±12.9 seeds per lesion (range, 12-63).

### Efficacy and local control

The follow-up period was 1-55 months (median 12 months). In patients with symptoms, the symptomatic relief was obtained in 86.95% (20/23) of patients. ^125^I brachytherapy resolved the symptoms associated with tumor infiltration including pain (5/5), frequent and urgent urination (4/5), tenesmus caused by rectum pressure (8/9), rectum or vaginal bleeding (3/4). The local control rate of 3, 6, 12, 24, and 36 months was 93.3%, 77.7%, 58.9%, 38.7%, 19.3%, respectively (Figure 2). At the time of writing, 3 patients presented good control of local tumor and no systemic recurrence throughout the 24 months follow-up period (Figure 4). According to Table 2, residual tumor size ≤1 (75.00% *vs* 36.40%, *P* = 0.002), tumor diameter ≤4cm (85.71% *vs* 40.00%, *P* = 0.026), no distant metastasis before the procedure (75.86% *vs* 44.44%, *P* = 0.031) and actual D90 between 110-130Gy (47.37% *vs* 66.67% *vs* 62.5%, *P* = 0.032) had significant association to better results in the univariate analysis. However, multivariate analysis identified only tumor diameter (HR 10.818, 95% CI 1.159-100.985; *P* = 0.037), and actual DVH parameter D90 (HR 0.129, 95% CI 0.021-0.808; *P* = 0.029) as independent prognostic factors associated with local control rate (Table 3).

**Figure 1:**
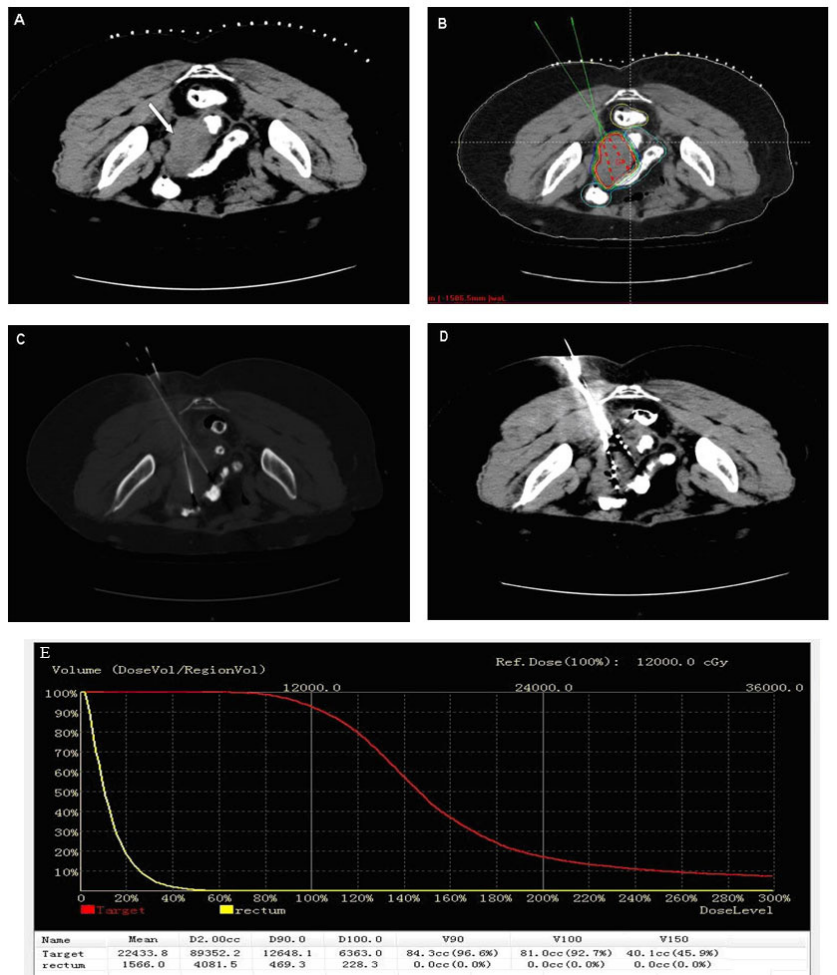
The administration of **^125^**I seeds brachytherapy. **A**. Preoperative transverse CT image was obtained for targeting area of interest. **B**. The isodose curve on treatment planning system. The position of brachytherapy applicator and the dose were calculated by treatment planning system. **C**. The implantation of applicators. **D**. The administration of ^125^I seeds. **E**. A dose-volume histogram of PTV and risk organ after seeds implantation.

**Figure 2:**
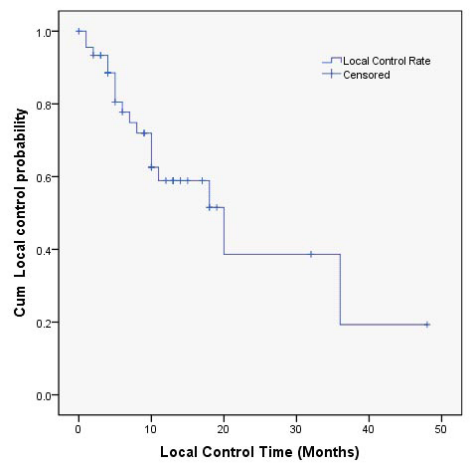
Local control probability after **^125^**I brachytherapy.

**Figure 3:**
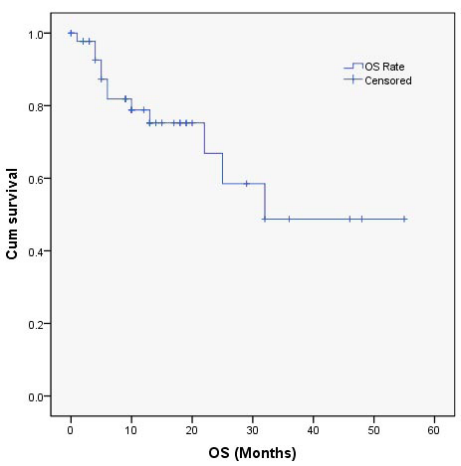
Overall survival after **^125^**I brachytherapy.

**Figure 4:**
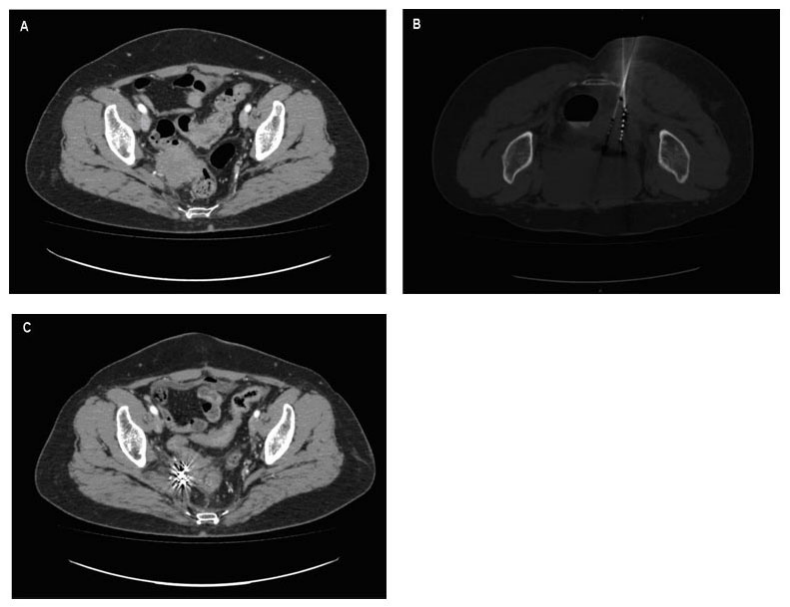
A case presentation **A**. Perirectal lesion recurred from ovarian cancer. **B**. During brachytherapy, an applicator accurately inserted into the tumor to implant ^125^I seeds. **C**. Four months after brachytherapy, tumor disappeared, with only well distributed radioactive seeds remaining.

**Table 1:** Patients’ characteristics (*n* = 47)

Parameter	Patients	
	No.	Percent (%)
Age, y		
Median (range)	53.3 (34-79)	
Performance status		
0	21	44.7
1	20	42.6
2	6	12.8
Pathological types		
Serous carcinoma	34	72.3
Endometrioid carcinoma	7	14.9
Mucinous carcinoma	2	4.3
Clear-cell carcinoma	3	6.4
Granulosa cell tumors	1	2.1
Tumor stage		
I-II	12	25.5
III-IV	35	74.5
Residual tumor size (cm)		
≤1	33	70.2
>1	14	29.8
The number of operation		
1	33	70.2
2	12	25.5
3	2	4.3
Site of the recurrence		
Vaginal stump	14	27.5
Perirectal	17	33.3
Pelvic sidewall	15	29.4
Para-aortic	5	9.8
Tumor diameter (cm)		
≤4	17	36.2
>4	30	63.8
Distant metastases before the procedure		
No	29	61.7
Yes	18	38.3
D90		
≤110	18	38.3
>110 to ≤130	21	44.7
>130	8	17.0
CA125 level (U/mL)		
≤35 >35	9 38	19.2 80.9

**Table 2:** Univariate analysis regarding local control rate (LC) and overall survival (OS)

Parameter	Category	*P* value (LC)	*P* value (OS)
Age (y)	≤50 vs >50	0.151	0.111
Performance status	0 vs 1vs 2	0.177	0.006
Pathological types	Serous carcinoma vs Endometrioid carcinoma Mucinous carcinoma vs Clear-cell carcinoma vs Granulosa cell tumors	0.250	0.865
Tumor stage	I-II vs III-IV	0.123	0.410
Residual tumor size (cm)	≤1 vs >1	0.002	0.049
Interval from the last chemotherapy(m)	≤6 vs >6	0.840	0.676
The number of operation	1 vs 2vs 3	0.892	0.463
The number of chemotherapy	≤8 vs >8	0.653	0.205
The frequency of recurrence	1 vs 2vs 3	0.186	0.188
Site of the recurrence	Vaginal stump vs Perirectal vs Pelvic sidewall vs Para-aortic	0.242	0.434
Tumor diameter (cm)	≤4 vs >4	0.026	0.085
distant metastases before the procedure	No vs Yes	0.031	0.016
D90	≤110 vs 110-130 vs >130	0.032	0.055
CA125 level (U/mL)	≤ 35 vs >35	0.671	0.910

**Table 3:** Multivariate Cox proportional analysis regarding local control rate (LC) and overall survival (OS)

Parameter	LC		OS	
	HR(95 % CI)	*P* value	HR(95 % CI)	*P* value
Performance status	/		3.821(1.383-10.555)	0.010
Residual tumor size (cm)	3.943(0.918-16.937)	0.065	6.157(1.438-26.367)	0.014
Tumor diameter (cm)	10.818(1.159-100.985)	0.037	/	
distant metastases before the procedure	4.148(0.991-17.363)	0.052	9.222(1.710-49.737)	0.010
D90	0.129(0.021-0.808)	0.029	/	

/: the *P* value >0.05 in univariate analysis regarding LC or OS.

### Overall survival

The mean OS of this study group was 14.6 months (Figure 3). In 12 patients who died, 5 cases died as a result of heart and lung function failure, 2 patients died of renal failure, and 5 cases died of extensive metastases. Better OS was found in patients who didn't have distant metastasis when received the ^125^I brachytherapy (*P* = 0.016), good performance status (*P* = 0.006) and small postoperative residual tumor size (*P* = 0.049) (Table 2). Age, ovarian cancer pathology, primary tumor stage, interval from the last chemotherapy, CA125 levels, the number of previous chemotherapy or operation, recurrence site, and ^125^I dosage had no association with OS. Based on the Cox proportional hazards regression analysis, no distant metastasis before the procedure (HR 9.222, 95% CI 1.710-49.737; *P* = 0.010), good performance status (HR 3.821, 95% CI 1.383-10.555; *P* = 0.010) and small postoperative residual tumor size (HR 6.157, 95% CI 1.438-26.367; *P* = 0.014) were still the independent predictors of better OS. The results are shown in Table 3.

### Distant metastases

Distant metastases were present in eighteen patients prior to ^125^I brachytherapy, including liver metastasis in 12 patients, spleen metastasis in 2 patients, liver and spleen metastasis in 1 patient, bone metastasis in 1 patient, and the other 2 patients with inguinal lymph node metastasis. In this study, the patients who had distant metastasis when received the ^125^I brachytherapy were correlated with poor OS (*P* = 0.010). Besides that, three patients developed distant metastases after the procedure. There had two liver metastasis (8 months and 14 months) and one lung metastasis (16 months) from the time of brachytherapy. The afterward metastasis had no association with OS (*P* = 0.894).

### Complications

No severe complications related to the ^125^I brachytherapy were observed (Table 4). In all, 28.8%(15/52) procedures experienced temporary minor side effects, including mild pain in the puncture area(8/52), small amount of vaginal bleeding(3/52) and gastrointestinal uncomfortable(4/52). These symptoms disappeared without treatment within1-2 weeks. Seeds migration occurred in 5/51 (9.8%) without any serious complications.

**Table 4:** Complications of 125I seed implantation

Complications	No. of Patients (%)
Pain	8(15.4)
vaginal bleeding	3(5.8)
gastrointestinal uncomfortable	4(7.7)
displacement of radioactive seeds	5(9.8)

## DISCUSSION

In this study, we demonstrated that ^125^I brachytherapy is an effective method to treat recurrent OC, especially for people with clinical symptoms caused by local tumor infiltration. This is important because it might be an alternative treatment for recurrent OC through this minimally invasive procedure. Despite the strong evidence for primary cytoreductive surgery in ovarian cancer, the evidence for surgery after disease relapse is less well defined. Second debulking surgery is not curative and it may confers a high risk of perioperative morbidity in some cases [16]. Chemotherapy is the usual treatment of recurrent OC, but its impact on quality of life remains uncertain [17]. Repeated administration of chemotherapy causes numerous intolerable cumulative toxicities, which worsen until continuation of chemotherapy finally becomes impossible. ^125^I brachytherapy provided an opportunity to achieve remission. As a low-dose rate brachytherapy, ^125^I seed emits continuous gamma rays, which can inhibit tumor cell mitosis and decrease the resistance effect of hypoxic cells to radiation [18, 19]. Brady et al [20] proved that ^125^I radiation could efficiently inhibit the proliferation and promote the apoptosis of tumor cells. Due to its long half-life (59.4 days) and low photon energy (27-35 KeV), ^125^I brachytherapy has increasingly been used for gynecological malignant tumors [21–23]. However, few institutions have reported slightly larger experiences with ^125^I brachytherapy for (recurrent) ovarian cancer.

With the recurrent rates of 70% or more, many OC patients will require further salvage therapies. For recurrent pelvic malignancies without evidence of systemic disease, the decision to add radiation to the salvage regimen should be considered [24–26]. As one kind of the radiotherapy, ^125^I brachytherapy has several dosimetric advantages when compared to the other kind of radiotherapy [18, 19, 22, 23]. Furthermore, the therapeutic benefit is theoretically boosted by natural increases in local dose after radiation-induced tumor shrinkage brings the ^125^I seeds closer together [27]. Despite these particular advantages, there are very limited reports about the important role of ^125^I brachytherapy in the treatment of OC. In our series, the local control rate of 3, 6, 12 and 24 months was 93.3%, 77.7%, 58.9%, 38.7%, respectively, and the patients released the symptom very quickly. Besides that, we found that patients with tumor size > 4 cm had significantly lower LC (*P* = 0.037). This is consistent with the other kind of radiotherapy [28]. However, FIGO-stage was not an independent prognostic factor in this study. It can be explained that the radiation technique used and enrolled indication criteria were different from this report. Some studies also indicated that special histopathology type such as clear cell carcinoma had a favorable prognosis of radiotherapy but we didn't find the relationship [29]. In our study, some patients were failed to respond to one or more drugs and the recurrence frequency was more than two times, which led to more complicated chemotherapy regimens and chemotherapy cycles. Of course, this was also an important reason for the patients in poor general condition, so that they could not accept other treatments. ^125^I brachytherapy is a local treatment and we have shown that performance status can not influence the LC. That indicates that the patient's condition does not affect the efficacy of ^125^I brachytherapy, and the symptoms associated with tumor infiltration are resolved significantly after ^125^I seed implantation.

Another important finding in our study is the dose-effect relationship in recurrent OC. With an actual D90 of 110-130Gy, a better LC rate was obtained. This is corresponding to the AAPM (American Association of Physicists in Medicine) recommendation that clinics reduce the prescribed dose for ^125^I implantation monotherapy from 160 to144Gy [30]. This recommendation dose is mainly for prostate carcinoma. When the prescribed dose reached up to this limit, serious side effects may occur. Although in this study group, we didn't had any serious side effects observed, we recommend the prescribed dose less than 130Gy with TPS plan. Physicians should be cautious when using a dose higher than 130Gy since no better LC was observed and severe complications may occur at this dose level. A treatment planning system can help peripheral tumor doses reach the matched peripheral dose [31]. It can make more than 95% of the tumor get 100% of the prescription dose without increasing radiation to surrounding healthy tissues. This satisfies the American Brachytherapy Society's so-called dual 90, which is a cancer cure requires that 90% of the tumor volume acquire 90% prescription dose [32]. With this in mind, a careful TPS should be done before the ^125^I brachytherapy for each patient and repeat DVH with close observation should be applied during the procedure.

^125^I seed local treatment can reduce the tumor burden, relieve local symptoms and improve quality of life of patients. But some patients had distant metastases before ^125^I seed implantation. Although distant metastases had been treated with other therapies, it still had an important influence on prognosis. The mean OS in this study was14.6 months. The overall survival rates of patients with distant metastases before the procedure were significantly lower 55.6% (10/18) than those with no distant metastasis 89.3% (25/28) (*P* = 0.010). By the time of writing this paper, the two patients who had longest OS were 55 and 48 months, respectively. All of them didn't have distant metastasis before the procedure. For the 12 death, 66.7% (8/12) patients had distant metastases prior to ^125^I brachytherapy. Furthermore, good performance status (*P* = 0.010) and small postoperative residual tumor size (*P* = 0.014) were closely correlated with a better OS. So, this reminds us that postoperative residual tumor size is an important predictor for the prognosis of patients with ^125^I brachytherapy.

The success of ^125^I brachytherapy for recurrent OC is dependent on the accurate placement of radioactive seeds [33]. The biggest concern in terms of dosimetric success is the elimination of pelvic seeds migration. In this study, 9.8% (5/51) patients were observed to have seeds migration at 3 months after the procedures. This phenomenon may contribute to the natural mixed cystic and solid structure of the ovarian cancer. Abhirup Sarkar et al. [34] found that although Day 0 implants (radiographs and CT scans obtained after implant) appeared quite conformal with no migration, by Day 30, thirty percent (3/10) had demonstrated significant pelvic shift in prostate brachytherapy. In another study, seeds migration also resulted in a higher dose to the urethra and rectal wall with a trend toward more acute rectal toxicity in prostate cancer brachytherapy [35]. Although it didn't induce any side effect in our study group, physicians should aware this phenomenon and a CT scan should be done not only at the end of the procedure but also thereafter to monitor the seeds migration and possible side effects.

Although it is difficult to compare differences in treatment techniques and subsequent outcomes, the patients received limited whole-pelvis [28]or electron-beam radiation [36]had more subsequent morbidity and mortality. Chronic bowel toxicity, particularly small bowel obstruction, is a potential problem in all patients receiving radiation therapy to the abdomen or pelvis. Compared with the complications related to other kind of radiotherapy, ^125^I brachytherapy appears more tolerable. Adverse events of adjuvant ^125^I implantation are generally related to puncture process, but are generally mild and manageable, such as pain of the puncture site and small amount of vaginal bleeding. Moreover, some patients will feel intestinal uncomfortable when we need to pass through the bowel during ^125^I implantation. But, the recovery of bowel function is quickly, and no serious damage to the intestinal. No procedure-related deaths or major complications occurred. Based on these results, ^125^I brachytherapy is an effective and safe modality for the treatment of recurrent ovarian cancer.

However, there are some limitations concerning the present study. This study was limited by its retrospective nature, and relatively small sample size from a single institution. More than 60% of the patients were within the recent two years and the follow up period were relatively short. Given the propensity of ovarian cancer to recur in the abdomen, ^125^I brachytherapy may be suitable for patients with isolated recurrences after resection and resistant to conventional chemotherapy. Fortunately, in this study, we found a good local control rate and patients tolerance of ^125^I brachytherapy. However, large-scale multicentre studies with long-term follow-up will be designed to confirm the efficacy and safety of ^125^I seeds for recurrent ovarian cancer in the future.

In conclusion, ^125^I brachytherapy is an effective and safe salvage therapy for patients in poor general condition with a previous history of various types of therapy, and may be the most appropriate treatment for recurrent tumors of small size (tumor volume of < 4cm) located within the pelvic. In a word, ^125^I brachytherapy for recurrent ovarian cancer is a promising area of investigation to improve local disease control and quality of life.

## MATERIALS AND METHODS

### Patients

This study was approved by institutional review board and was performed in compliance with hospital ethics and clinical practice guidelines. The study included patients who with recurrent ovarian cancer. In addition, all patients were medically inoperable or patients and their families refused surgery and failed to respond to one or more chemotherapeutic regimens. Patients were excluded from the study if they had (1) uncorrectable coagulopathy (international normalized ratio > 1.5), (2) thrombocytopenia (platelet count < 50,000/L), (3) severe history of mental disease, (4) severe renal or hepatic function impairment.

A total of 47 patients (median 53.3 years, range, 34-79 years) with 51 recurrent OC lesions received ^125^I brachy therapy from March 2011 to August 2015 were enrolled in this study. The patients distribution in 2011 to 2015 was 6, 6, 5, 17, 13; respectively. 74.5% (35/47) were FIGO stage III-IV; the mean size of the lesions was 5.14 cm ± 1.65 (range, 2.8-7.3cm) in the largest diameter. As to the recurrent sites, 27.5%(14/51) were at the vaginal stump, 9.8% (5/51) were para-aortic, 33.3% (17/51) were around the rectum, 29.4% (15/51) were irregular masses extended laterally to the pelvis. The mean value of CA125 was 546.13u/ml (range, 3.4 - 6800 u/ml). All patients had received surgical cytoreduction and 29.78 % (14/47) had more than one times of surgery. The mean time of the lesions were seen on images was 29.1 months (range, 2-168months) after the last resection of the primary cancer. The diagnosis of recurrent OC was confirmed with pathologic proof at CT-guided needle biopsy in all patients. In all patients, 72.3% (34/47) were serous carcinoma, 14.9% (7/47) were endometrioid carcinoma, 4.3% (2/47) were mucinous carcinoma, 6.4% (3/47) were clear cell carcinoma and 2.1% (1/47) were granular cell carcinoma. The patients had previously been treated with a median of 3 chemotherapeutic agents (range, 2-11) over a median of 12 treatment cycles (range, 1-30). Patient characteristics are listed in Table 1.

### ^125^I brachytherapy treatment planning

The brachytherapy treatment planning system (TPS, Beijing Aerospace and Aviation University, Beijing, China) was used to create implant plans based on patients’ CT images (Figure 1a). The prescribed dose target volume (PTV) was outlined by oncologists to cover the lesion with a 0.5-1.0 cm margin. The prescribed dose (PD) of the ^125^I implant was 90-150Gy, which was adjusted according to the adjacent structures. PTV edge was covered by isodose curve from 80% to 90% (Figure 1b). Dose-volume histogram (DVH), isodose curves of different percentages, position of brachytherapy applicator and number of implanted seeds were generated (Figure 1c). Implantation of ^125^I seeds ( model 6711, 4.5 mm long and 0.8 mm in diameter; radioactivity, 0.6-0.8 mCi; average energy, 27-35 keV; half- life of 59.6 days; antitumor activity, 1.7 cm; initial dose rate, 7 cGy/h; China Institute of Atomic High Tech) was performed under CT guidance according to the plan (Figure 1d, 1e).

### ^125^I brachytherapy procedure

All the ^125^I seeds implantation was performed in a standard CT room under local anesthesia and CT imaging was taken at intervals of 5 mm. With CT fluoroscopic guidance, 18 G implantation needles were inserted into target lesions avoiding puncture of large blood vessels and nearby important organs, a turntable gun was then used to place ^125^I seeds into recurrent tumors and seeds were released 0.5-1cm apart upon withdrawing the needles. However, those close to critical structures such as vital blood vessels, intestine and bladder, ^125^I seeds must be kept at least one centimeter away. The evaluation of post plan was routinely obtained for each patient. For tumors showing insufficient radioactivity, more ^125^I seeds were implanted.

### Follow-up

Follow-up consisted of record of symptoms improvement, routine physical examinations and appropriate imaging examination. A CT scan was undertaken 1 and 3 months after the procedure, and then at 3-month intervals after implantation, or as necessary. The objective response rate of ^125^I brachytherapy was calculated according to the Response Evaluation Criteria in Solid Tumors (RECIST) [14]. Local control rate (LC) was defined as the proportion of patients who received complete response and partial response according to RECIST. The overall survival (OS) was calculated from the date of implantation to the final follow-up assessment or the date of death. In our research, the definition of distant metastasis refers to metastasis outside the abdomen, for example, liver metastasis, lung metastasis, spleen metastasis, and so on. The presence of distant metastases was checked using X-rays, ultrasound, CT or PET-CT. Complications were evaluated according to the Radiation Therapy Oncology Group (RTOG)/European Organization for Research and Treatment of Cancer (EORCT) grading system [15].

### Statistical analysis

Data collected were analyzed using SPSS version 16.0 (SPSS Inc., Chicago, IL). The probabilities of LC and OS were calculated using the Kaplan-Meier product-limit method. Possible predictive factors were analyzed for impact on LC and on OS with univariate analyses using the classical log-rank test. Cox proportional hazards model was used for multivariate analysis. A two-sided *P* < 0.05 was considered statistical significant.

## References

[1] Siegel RL, Miller KD, Jemal A (2015). Cancer statistics, 2015. CA Cancer J Clin.

[2] Classe JM, Jaffre I, Frenel JS, Bordes V, Dejode M, Dravet F, Ferron G, Marchal F, Rigaud DB, Loussouarn D, Campion L (2011). Prognostic factors for patients treated for a recurrent FIGO stage III ovarian cancer: a retrospective study of 108 cases. Eur J Surg Oncol.

[3] Polcher M, Zivanovic O, Chi DS (2014). Cytoreductive surgery for advanced ovarian cancer. Womens Health (Lond Engl).

[4] Thigpen T (2012). A rational approach to the management of recurrent or persistent ovarian carcinoma. Clin Obstet Gynecol.

[5] Jordan GH, Lynch DF, Warden SS, McCraw JD, Hoffman GC, Schellhammer PF (1985). Major rectal complications following interstitial implantation of 125iodine for carcinoma of the prostate. J Urol.

[6] Mohler JL, Armstrong AJ, Bahnson RR, Boston B, Busby JE, D’Amico AV, Eastham JA, Enke CA, Farrington T, Higano CS, Horwitz EM, Kantoff PW, Kawachi MH (2012). Prostate cancer, Version 3.2012: featured updates to the NCCN guidelines. J Natl Compr Canc Netw.

[7] Ostertag CB, Kreth FW (1995). Interstitial iodine-125 radiosurgery for cerebral metastases. Br J Neurosurg.

[8] Horwitz EM, Frazier AJ, Martinez AA, Keidan RD, Clarke DH, Lacerna MD, Gustafson GS, Heil E, Dmuchowski CF, Vicini FA (1996). Excellent functional outcome in patients with squamous cell carcinoma of the base of tongue treated with external irradiation and interstitial iodine 125 boost. Cancer.

[9] Wang G, Zhang F, Yang B, Xue J, Peng S, Zhong Z, Zhang T, Lu M, Gao F (2015). Feasibility and Clinical Value of CT-guided I Brachytherapy for Bilateral Lung Recurrences from Colorectal Carcinoma. Radiology.

[10] Trombetta MG, Colonias A, Makishi D, Keenan R, Werts ED, Landreneau R, Parda DS (2008). Tolerance of the aorta using intraoperative iodine-125 interstitial brachytherapy in cancer of the lung. Brachytherapy.

[11] Nag S, DeHaan M, Scruggs G, Mayr N, Martin EW (2006). Long-term follow-up of patients of intrahepatic malignancies treated with iodine-125 brachytherapy. Int J Radiat Oncol Biol Phys.

[12] Wang H, Wang J, Jiang Y, Li J, Tian S, Ran W, Xiu D, Gao Y (2013). The investigation of 125I seed implantation as a salvage modality for unresectable pancreatic carcinoma. J Exp Clin Cancer Res.

[13] Kim JH, Hilaris B (1975). Iodine 125 source in interstitial tumor therapy Clinical and biological considerations. Am J Roentgenol Radium Ther Nucl Med.

[14] Therasse P, Arbuck SG, Eisenhauer EA, Wanders J, Kaplan RS, Rubinstein L, Verweij J, Van Glabbeke M, van Oosterom AT, Christian MC, Gwyther SG (2000). New guidelines to evaluate the response to treatment in solid tumors European Organization for Research and Treatment of Cancer, National Cancer Institute of the United States, National Cancer Institute of Canada. J Natl Cancer Inst.

[15] Cox JD, Stetz J, Pajak TF (1995). Toxicity criteria of the Radiation Therapy Oncology Group (RTOG) and the European Organization for Research and Treatment of Cancer (EORTC). Int J Radiat Oncol Biol Phys.

[16] Eisenkop SM, Friedman RL, Spirtos NM (2000). The role of secondary cytoreductive surgery in the treatment of patients with recurrent epithelial ovarian carcinoma. Cancer.

[17] Song YS, Kim HS, Aoki D, Dhanasekaran DN, Tsang BK (2014). Ovarian cancer. Biomed Res Int.

[18] DeWeese TL, Shipman JM, Dillehay LE, Nelson WG (1998). Sensitivity of human prostatic carcinoma cell lines to low dose rate radiation exposure. J Urol.

[19] Koritzinsky M, Wouters BG, Amellem O, Pettersen EO (2001). Cell cycle progression and radiation survival following prolonged hypoxia and re-oxygenation. Int J Radiat Biol.

[20] Yang G, Peng S, Zhang Y, Liu Z, Lu M, Zhang T, Gao F, Zhang F (2014). Cell-based assay system to estimate the effect of 125I seeds on cancer cells: effect of osteopontin. Recent Pat Anticancer Drug Discov.

[21] Monk BJ, Tewari KS, Puthawala AA, Syed AM, Haugen JA, Burger RA (2002). Treatment of recurrent gynecologic malignancies with iodine-125 permanent interstitial irradiation. Int J Radiat Oncol Biol Phys.

[22] Sharma SK, Forgione H, Isaacs JH (1991). Iodine-125 interstitial implants as salvage therapy for recurrent gynecologic malignancies. Cancer.

[23] Brady LW, Micaily B, Miyamoto CT, Heilmann HP, Montemaggi P (1995). Innovations in brachytherapy in gynecologic oncology. Cancer.

[24] Guren MG, Undseth C, Rekstad BL, Braendengen M, Dueland S, Spindler KL, Glynne-Jones R, Tveit KM (2014). Reirradiation of locally recurrent rectal cancer: a systematic review. Radiother Oncol.

[25] Sole CV, Calvo FA, Lozano MA, Gonzalez-Bayon L, Gonzalez-Sansegundo C, Alvarez A, Lizarraga S, Garcia-Sabrido JL (2014). External-beam radiation therapy after surgical resection and intraoperative electron-beam radiation therapy for oligorecurrent gynecological cancer. Long-term outcome Strahlenther Onkol.

[26] Backes FJ, Martin DD (2015). Intraoperative radiation therapy (IORT) for gynecologic malignancies. Gynecol Oncol.

[27] Pouliot J, Tremblay D, Roy J, Filice S (1996). Optimization of permanent 125I prostate implants using fast simulated annealing. Int J Radiat Oncol Biol Phys.

[28] Fujiwara K, Suzuki S, Yoden E, Ishikawa H, Imajo Y, Kohno I (2002). Local radiation therapy for localized relapsed or refractory ovarian cancer patients with or without symptoms after chemotherapy. Int J Gynecol Cancer.

[29] Macrie BD, Strauss JB, Helenowski IB, Rademaker A, Schink JC, Lurain JR, Small W (2014). Patterns of recurrence and role of pelvic radiotherapy in ovarian clear cell adenocarcinoma. Int J Gynecol Cancer.

[30] Williamson JF, Butler W, Dewerd LA, Huq MS, Ibbott GS, Mitch MG, Nath R, Rivard MJ, Todor D, American Association of Physicists in Medicine (2005). Recommendations of the American Association of Physicists in Medicine regarding the impact of implementing the 2004 task group 43 report on dose specification for 103Pd and 125I interstitial brachytherapy. Med Phys.

[31] Pignol JP, Rakovitch E, Keller BM, Sankreacha R, Chartier C (2009). Tolerance and acceptance results of a palladium-103 permanent breast seed implant Phase I/II study. Int J Radiat Oncol Biol Phys.

[32] Nag S (2000). Brachytherapy for prostate cancer: summary of American Brachytherapy Society recommendations. Semin Urol Oncol.

[33] Nath R, Anderson LL, Luxton G, Weaver KA, Williamson JF, Meigooni AS (1995). Dosimetry of interstitial brachytherapy sources: recommendations of the AAPM Radiation Therapy Committee Task Group No 43. American Association of Physicists in Medicine. Med Phys.

[34] Sarkar A, Donavanik V, Zhang I, Chen H, Koprowski C, Hanlon A, Mourtada F, Strasser J, Raben A (2013). Prostate implant dosimetric outcomes and migration patterns between bio-absorbable coated and uncoated brachytherapy seeds. Brachytherapy.

[35] Saibishkumar EP, Borg J, Yeung I, Cummins-Holder C, Landon A, Crook JM (2008). Loose seeds vs. stranded seeds: a comparison of critical organ dosimetry and acute toxicity in (125)I permanent implant for low-risk prostate cancer. Brachytherapy.

[36] Sole CV, Calvo FA, Lizarraga S, Gonzalez-Bayon L, Garcia-Sabrido JL (2015). Intraoperative electron-beam radiation therapy with or without external-beam radiotherapy in the management of paraaortic lymph-node oligometastases from gynecological malignancies. Clin Transl Oncol.

